# Clinical and radiological correlates of activities of daily living in cerebellar atrophy caused by *PMM2* mutations (PMM2-CDG)

**DOI:** 10.1007/s12311-021-01242-x

**Published:** 2021-02-22

**Authors:** Fabio Pettinato, Giovanni Mostile, Roberta Battini, Diego Martinelli, Annalisa Madeo, Elisa Biamino, Daniele Frattini, Domenico Garozzo, Serena Gasperini, Rossella Parini, Fabio Sirchia, Giuseppe Sortino, Luisa Sturiale, Gert Matthijs, Amelia Morrone, Maja Di Rocco, Renata Rizzo, Jaak Jaeken, Agata Fiumara, Rita Barone

**Affiliations:** 1 Child Neurology and Psychiatry Section, Department of Clinical and Experimental Medicine, University of Catania, Policlinico, Via Santa Sofia 78, 95123 Catania, Italy; 2 Department “GF Ingrassia”, Section of Neurosciences, University of Catania, Catania, Italy; 3 Department of Developmental Neuroscience, IRCCS Stella Maris Foundation, Pisa, Italy; 4 Department of Clinical and Experimental Medicine, University of Pisa, Pisa, Italy; 5 Division of Metabolism, Department of Pediatric Specialties, Bambino Gesù Children’s Hospital, IRCCS, Rome, Italy; 6 Unit of Rare Diseases, IRCCS Istituto Giannina Gaslini, Genoa, Italy; 7 Department of Pediatrics, University of Turin, Turin, Italy; 8 Department of Pediatrics, Child Neurology Unit, Presidio Ospedaliero Provinciale Santa Maria Nuova Azienda USL-IRCCS di Reggio Emilia, Reggio Emilia, Italy; 9 CNR, Institute for Polymers, Composites and Biomaterials, IPCB, Catania, Italy; 10 Pediatric Rare Diseases Unit, Department of Pediatrics, MBBM Foundation, ATS Monza e Brianza, Monza, Italy; 11 Department of Molecular Medicine, University of Pavia, Pavia, Italy; 12 Department of Diagnostic Imaging, Radiology Unit, Policlinico University Hospital, Catania, Italy; 13 Department of Human Genetics, KU Leuven, Leuven, Belgium; 14 Molecular and Cell Biology Laboratory of Neurometabolic Diseases, Neuroscience Department, Meyer Children’s Hospital, Florence, Italy; 15 Department of NEUROFARBA, University of Florence, Florence, Italy; 16 Department of Development and Regeneration, Centre for Metabolic Diseases, University Hospital Gasthuisberg, KU Leuven, Leuven, Belgium; 17 Pediatric Unit, Regional Referral Center for Inherited Metabolic Disease, University of Catania, Catania, Italy

**Keywords:** Congenital disorder(s) of glycosylation, *PMM2* variants, Cerebellar atrophy, Pons atrophy, Ataxia, Activities of daily living

## Abstract

We aimed to identify clinical, molecular and radiological correlates of activities of daily living (ADL) in patients with cerebellar atrophy caused by *PMM2* mutations (PMM2-CDG), the most frequent congenital disorder of glycosylation. Twenty-six PMM2-CDG patients (12 males; mean age 13 ± 11.1 years) underwent a standardized assessment to measure ADL, ataxia (brief ataxia rating scale, BARS) and phenotype severity (Nijmegen CDG rating scale, NCRS). MRI biometry of the cerebellum and the brainstem were performed in 23 patients (11 males; aged 5 months–18 years) and 19 control subjects with equal gender and age distributions. The average total ADL score was 15.3 ± 8.5 (range 3–32 out of 36 indicating severe functional disability), representing variable functional outcome in PMM2-CDG patients. Total ADL scores were significantly correlated with NCRS (*r*^*2*^ = 0.55, *p* < 0.001) and BARS scores (*r*^*2*^ = 0.764; *p* < 0.001). Severe intellectual disability, peripheral neuropathy, and severe *PMM2* variants were all significantly associated with worse functional outcome. Higher ADL scores were significantly associated with decreased diameters of cerebellar vermis (*r*^*2*^ = 0.347; *p* = 0.004), hemispheres (*r*^*2*^ = 0.436; *p* = 0.005), and brainstem, particularly the mid-pons (*r*^*2*^ = 0.64; *p* < 0.001) representing the major radiological predictor of functional disability score in multivariate regression analysis. We show that cerebellar syndrome severity, cognitive level, peripheral neuropathy, and genotype correlate with ADL used to quantify disease-related deficits in PMM2-CDG. Brainstem involvement should be regarded among functional outcome predictors in patients with cerebellar atrophy caused by PMM2-CDG.

## Introduction

Congenital disorders of glycosylation (CDG) are caused by pathogenic variants in the genes coding for proteins involved in the assembly and remodelling of the oligosaccharide moieties of glycoproteins and glycolipids. The list of CDG, mainly encompassing the protein N-linked and O-linked glycosylation pathways, is rapidly growing [1]. The protein N-linked CDG have been divided into CDG-I (glycan assembly in the cytosol and the endoplasmic reticulum) and CDG-II (glycan remodelling in the Golgi). Phosphomannomutase 2 (PMM2) enzyme deficiency (PMM2-CDG) (OMIM 212065) is the most frequent N-glycosylation defect with an estimated incidence of 1:20000 [2]. PMM2 is required in the early steps of the N-glycosylation pathway, and its deficiency is associated with hypoglycosylation of numerous serum and membrane glycoproteins [1, 2]. PMM2-CDG is a multisystem disease with a broad neurological phenotype including cerebellar hypotrophy/atrophy, psychomotor delay/intellectual disability, acquired microcephaly, strabismus and retinopathy, epilepsy, stroke-like episodes and peripheral neuropathy [3]. Hyperkinetic movement disorders such as dystonia and choreo-athetosis can also be part of this spectrum [4]. Cerebellar syndrome is a predominant feature and the main cause of disability in these patients [2, 5]. An early cerebellar sign is unusual jerky, conjugate oscillation of the eyes [6], while at a later stage, truncal titubation when sitting, dysmetria, slurred speech (dysarthria) and gait ataxia become more evident. Cerebellar ataxia has been reported from 96.7% [7] to 100% [8–10], and only a minority of patients is able to walk without support [2, 8, 9, 11]. Cerebellar size reduction in PMM2-CDG is thought to be the result of both hypoplasia and hypotrophy. There is a major involvement of the anterior lobe of the vermis, enlargement of cerebellar fissures, and the fourth ventricle often associated with cortical hyperintensity (“bright cerebellum”) and mildly hyperintense subcortical white matter [12].

Potential therapies and clinical trials have been emerging prompting the search of accurate outcome measures [13, 14], including an assessment of activities of daily living (ADL). The subscale ADL from the Friedreich Ataxia Rating Scale (FARS) [15] is a functional measurement that has been used in children with congenital cerebellar atrophy [16]. We measured ADL in a cohort of patients with PMM2-CDG and report here the prevalence of disability related to ADL in this sample. In order to delineate possible clinical and radiological correlates of functional disability, we considered possible associations among ADL, patients’ genotype and associated neurological features. In particular, the impact of cerebellar and brainstem biometry on functional outcome measured by ADL was investigated.

## Methods

### Participants

Twenty-six Italian subjects with a molecular diagnosis of PMM2-CDG were evaluated between January 2017 and November 2019. Patients were followed at our centre (*n*: 12) and academic medical centres spread over the country. Written informed consent was obtained from all participants and/or their relatives for enrolment in a national multicentre, cross-sectional study conducted in accordance with the internal institutional ethical committee procedures at the Child Neuropsychiatry Unit, University Hospital Catania, Italy. The procedures performed were in accordance with the principles of the 1964 Declaration of Helsinki and its later amendments (2013). Clinical data including family history, symptoms at onset, findings at physical examination and neurological examination, cognitive level, presence of epilepsy, stroke-like episodes and/or peripheral neuropathy, electroclinical features and neuroimaging were collected as part of the routine clinical care of the patients. The *PMM2* genotype was determined in each patient. *PMM2* allelic variants were classified as severe (loss-of-function mutations) or mild (missense mutations) according to published expression studies and enzymatic residual activity [17].

### Standardized Outcome Measures

#### Nijmegen CDG rating scale

The Nijmegen CDG rating scale (NCRS) is a validated tool to assess clinical characteristics and current functions of patients with CDG [18]. The NCRS includes information on three different sections (I–III): current function, system-specific involvement and current clinical assessment. We collected information from all sections with special regard to the current function section (section I) that is based on an interview with the patient or caregiver regarding vision, hearing, communication, feeding, mobility and educational achievements over the last 4 weeks. [18]. For each item, there are four possible responses reflecting normal (0), mild (1), moderate (2) and severe (3) impairment.

#### Brief Ataxia Rating Scale

In patients older than 10 years, severity of the cerebellar syndrome was assessed by the “Brief Ataxia Rating Scale” (BARS), a shorter modified form of the “International Cooperative Ataxia Rating Scale” (ICARS) [19]. Five major domains such as gait, upper and lower limb coordination, speech and ocular coordination were investigated. Scores ranged from 0 (absence of cerebellar symptoms) to 30 (severe ataxia). Total score was obtained based on the evaluation of gait (maximum score of 8), knee-tibia test and finger-to-nose test (maximum score of 4 for each limb), dysarthria (maximum score of 4) and oculomotor abnormalities (maximum score of 2). Since BARS scores are age-dependent in children up to 11 years [20] and, because most patients with PMM2-CDG had severe developmental delay, in patients with age < 10 years, the impact of the cerebellar syndrome on motor function was quantified on the NCRS current function section (mobility scores: from 0 (age appropriate mobility) to 3 (wheelchair dependent)).

### Activities of Daily Living

Quantification of daily life activities was computed by measuring nine domains of ADL: speech, swallowing, ability to feed itself, dressing, sitting, walking, frequency of falls, self-hygiene and bladder function. Scores ranged from 0 (normal function) to 4 (severe functional disability), with the maximum overall score of 36 indicating very severe functional disability [15, 16]. ADL scores were attributed through careful observation of the patient and after an exhaustive interview with his/her parents, especially the primary caregiver.

### Magnetic Resonance Imaging Studies

**Fig. 1:**
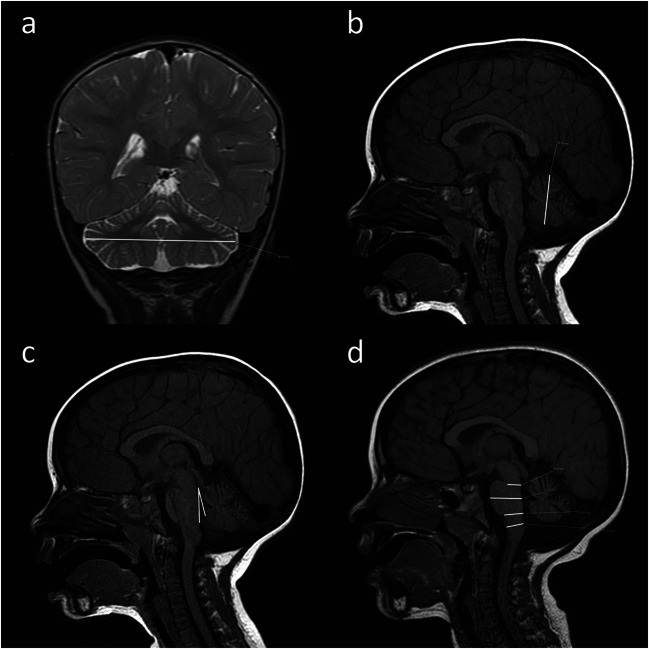
Biometry shown on brain MRI of a 7-year-old child with PMM2-CDG. **a** Maximum transverse cerebellar diameter (MCD). **b** Maximum craniocaudal height of the vermis (CCD). **c** Tegmento-vermian angle (TVA). **d** Anteroposterior measurements of the brainstem (**a** T2-weighted coronal image; **b–d** midline T1-weighted sagittal images)

Biometry of the cerebellum and brainstem was performed on brain magnetic resonance imaging (MRI) from 23 PMM2-CDG patients aged 5 months to 18 years (measurement unit: cm). Normal brain MRIs from 19 subjects group-matched by gender and age with study patients were collected, to set internal reference values for statistical comparative analyses. Control subjects for the MRI measurements were selected from the Catania University Hospital neuroimaging database. Control MRI studies and measurements were obtained following the same protocol. Control patients underwent MRI studies due to headache (*n* = 5), generalized idiopathic epilepsy (*n* = 5), a tic disorder (*n* = 5) or a minor head trauma (*n* = 4). All examinations included sagittal T1-weighted and axial/coronal T2-weighted images and fluid-attenuated inversion recovery (FLAIR) sequences. Biometry of the cerebellar hemispheres and vermis and brain stem structures were obtained for defining the degree of atrophic changes. Cerebellar hemispheres were measured by using the maximum transverse cerebellar diameter (MCD) on coronal T2-weighted images compared to the maximum diameter of the posterior cranial fossa (MFD) [21] (Fig. 1a). To assess the cerebellovermian biometry, the craniocaudal diameter (CCD) (maximum vermis craniocaudal height) was measured from the tip of the culmen lobule to the pyramid lobule on sagittal, midline MRI T1-weighted (Fig. 1b). The tegmento-vermian angle (TVA) was measured between a first line drawn parallel to the tegmentum of the brainstem and a second line drawn along the anterior aspect of the vermis (Fig. 1c). The TVA has been described as a way of measuring the “closure” of the fourth ventricle, which can be roughly considered a consequence of vermis growth, so that a significantly elevated angle is often associated with vermian and cerebellar hypoplasia [22].

Four anteroposterior measurements of the brainstem were performed on sagittal, midline T1-weighted images, at the junction of the mesencephalon and pons (mesencephalon), at the mid-pons (pons), at the pontomedullary junction (medulla) and at clava, as shown (Fig. 1d).

### Statistical Analysis

Scalar measures are presented as mean ± standard deviation; range and/or 95% confidence interval (95%CI). For categorical data frequency and percent values are reported. After testing for normal distribution, differences in scalar measures between groups were tested using the independent-samples *t*-test. Correlation analysis was performed by computing the coefficient of determination *R*^*2*^.

A multiple linear regression model was constructed using ADL total score as dependent variable and significantly correlated MRI parameters at univariate analysis, including age at MRI as a confounding variable *a priori*. We used a stepwise backward selection method, setting a significance level to 0.1 to identify major significant predictors for the outcome measure. Statistical variation in the dependent variable accounted for by the model was tested using the ANOVA test. Correlation between observed and predicted values was obtained computing the multiple correlation coefficients *R*^*2*^. Significance α-level was set at 0.05.

## Results

### Demographic and Clinical Characteristics

Twenty-six Italian patients with PMM2-CDG (12 males and 14 females, mean age 13 ± 11.1 years) from 24 unrelated families were included. Thirteen (53%) were children younger than 10 years (mean age 5.5 ± 2.6 years). In all patients, PMM2-CDG was diagnosed following the detection of developmental delay, dysmorphism (i.e. facial dysmorphism, inverted nipples, fat pads, lipodystrophy and increased internipple distance) and cerebellar atrophy at MRI. In patients with a severe phenotype, the mean age at diagnosis was 12 ± 0.4 months, although the two original patients were diagnosed in adulthood owing to the detection of olivopontocerebellar atrophy in the context of congenital cerebellar ataxia [23]. Early clinical features included hypotonia, strabismus, acquired microcephaly, feeding problems and extra-neurological signs such as enteropathy and recurrent increase of serum transaminases. Ten subjects (38%) had the *PMM2* missense mutations p.L32R (*n*: 6) and p. C241S (*n*: 4), associated with a milder neurological phenotype [8]. These patients were diagnosed at a later age (mean 7 ± 7.9 years), and in most of them, the presenting symptoms were developmental delay/disability and cerebellar signs (ataxia).

At the time of the study, all patients had intellectual disability of variable degree. Almost half of PMM2-CDG participants had full intelligence quotient (IQ) indicating borderline: mild to moderate ID (53%). In the remaining patients (46%), IQ level could not be measured because of severe to profound intellectual disability. Additional neurological symptoms included hypotonia (100% of patients), peripheral neuropathy (53%), extra-pyramidal signs (dystonia and dyskinesia) (42%), abnormal mood or behaviour and stroke-like episodes (32% each), epilepsy (myoclonic, absence and tonic-clonic seizures) (23%) and pyramidal signs (16%). Fifteen patients (56%) were unable to walk independently and depended on a wheelchair. Coagulation problems (68%), hepatic dysfunction (52%) and endocrine disorders (42%) were the most common systemic findings.

### Cerebellar Syndrome

All patients presented cerebellar ataxia of variable degree. In patients aged ≥ 10 years (13 patients, 6 males and 7 females, with a mean age 22.5 ± 11.2 years), the average total BARS score was 15.3 ± 8.2. Patients with severe *PMM2* allelic variants presented significantly higher BARS scores (24.2 ± 3.5) than those with milder variants (10.6 ± 5.4) (*p* < 0.001). BARS scores were also higher in patients with peripheral neuropathy by 13.3 points (95%CI 14.9–26.3) (*p* = 0.01). In children younger than 10 (13 patients, 7 males and 6 females, with a mean age 5.5 ± 2.6 years), the impact of the cerebellar syndrome on motor function was quantified by the mobility score on the NCRS. Among these patients, ten (77%) reported mobility scores ranging from 2 to 3 indicating that they could walk with support (*n*: 6) or were wheelchair dependent (*n*: 4), respectively. In 3 children (23%) with milder genotypes, NCRS mobility scored between 0 and 1, indicating age-appropriate mobility (*n*: 1) or mild difficulties (*n*: 2), respectively.

### Current Functions Measurement

Total ADL score (sum of nine ADL domains) was higher indicating more severe impairment. For analytical purposes, specific domains such as ADL speech and ADL self-care scores (sum of cutting food/handling utensils, dressing and personal hygiene) were also considered (Table 1). The average total ADL score was 15.3 ± 8.5 (range 3–32) out of a maximum of 36. Ten patients had total ADL higher than 20 (range 21–32); the most affected ADL areas were speech, walking and self-care (Table 1). The mean total of all elements of NCRS current function was 8.2 ± 4.1 on a maximum of 21. Total ADL and NCRS scores were significantly correlated (*r*^*2*^ = 0.55, *p* < 0.001).

**Table 1:** Demographic characteristics and standardized measures in patients with PMM2-CDG

	*n*	Mean	SD	Range	Reference values
Age at study time (years)	26	13.0	11.1	2–45	-
BARS total^a^	13	15.8	8.2	7–30	0–30
ADL					
Walking	26	2.32	1.3	0–4	0–4^b^
Speech	26	2.2	1.3	0–4	0–4
Self-care	21	7.2	3.5	1–12	0–12
Total^b^	26	15.3	8.5	3–32	0–36
NCRS					
Mobility	23	1.9	1.1	0–3	0–3
Communication	23	1.6	0.8	0–3	0–3
Self-care	23	1.9	1	0–3	0–3
Total^c^	23	24	10.4	10–47	Mild (0–14) Moderate (15–25) Severe (> 25)

^a^Brief ataxia rating scale (BARS) total scores 0–30: normal to severe ataxia

^b^Activities of daily living (ADL) assessed with appropriate regard for age (normal to severe functional disability). ADL total scores 0–36 ranked least to most affected

ADL self-care (sum of personal hygiene, dressing, utensil use, e.g. for feeding)

^c^Nijmegen CDG rating scale (NCRS) (assessed with appropriate regard for age). For each item, there are four possible responses reflecting normal (0), mild (1), moderate (2) and severe (3) impairment. NCRS total scores indicate global disease burden (including sections I, II and III)

Total ADL score was significantly higher in patients with higher BARS total score; thus, patients with severe ataxia presented with a more severe functional disability (*r*^*2*^ = 0.764; *p* < 0.001). Moreover, ADL score differences were computed between patient subgroups based on genotype, intellectual disability (ID) level (mild-moderate or severe) and clinical characteristics such as presence of epilepsy, peripheral neuropathy, extra-pyramidal signs and stroke-like episodes (Table 2). Total ADL score was higher in patients with severe genotypes than in those with milder allelic variants (ADL mean difference 12.5; 95%CI 11.1–13.9) (*p* < 0.001). Moreover, patients with severe ID had a significantly higher total ADL score compared to patients with mild-moderate cognitive impairment (mean difference 12.5; 95%CI 11.1–13.9) (*p* < 0.001).

**Table 2:** ADL score differences between PMM2-CDG clinical subgroups

Group	*n*	ADL mean (SD)	95%CI	ADL mean difference (95%CI)	*p* Value
Genotype*					
Mild	12	9.8 (1.4)	6.8–12.7		< 0.001
Severe	14	22.3 (1.9)	17.8–26.7	12.5 (11.1–13.9)	
Intellectual disability					
Mild-moderate	14	9.8 (5.1)	7.1–12.5		< 0.001
Severe	12	22.8 (6.5)	19.1–26.5	13 (8.3–17.7)	
Epilepsy					
Absent	18	13.1 (1.7)	9.6–16.7		0.057
Present	6	21.8 (4.2)	11.1–32.6	8.7 (6.3–11.1)	
Stroke-like episodes					
Absent	16	15.7 (1.9)	11.6–19.7		0.729
Present	6	17.2 (4.7)	4.9–29.4	1.5 (-1.4–4.4)	
Extra-pyramidal signs					
Absent	11	13.5 (1.8)	9.6–17.5		0.171
Present	11	18.6 (3.1)	11.7–25.6	5.1 (2.8–7.3)	
Neuropathy					
Absent	8	8.1 (1.2)	5.1–11.1		< 0.001
Present	14	20.6 (1.9)	16.5–24.8	12.5 (10.9–14.1)	

*ADL* activities of daily living. *PMM2 mild/severe allelic variants

ADL scores 0–36 ranked least to most affected

Six patients had epilepsy. In five subjects, seizures occurred on average once per month. One patient (p.V129M/R141H) showed more than 5 generalized tonic-clonic seizure episodes per month. All patients with epilepsy were treated with at least one antiepileptic drug at the time of the study. Total ADL scores were higher in participants with seizures than in those without seizures (ADL mean difference 8.7; 95%CI 6.3–11.1) with a borderline but non-significant difference (*p* = 0.057). Peripheral neuropathy was ascertained in 14 participants. Total ADL scores were significantly higher in the presence of peripheral neuropathy (ADL mean difference 12.5; 95%CI 10.9–14.1) (*p* < 0.001). No significant differences were observed with regard to the presence of extra-pyramidal signs and stroke-like episodes.

### MRI Biometry

Mean age at MRI study was 7.9 ± 6.2 years and 6.2 ± 5.4 years for the patient group and the control group, respectively (*p* = 0.375). Differences of cerebellar and brainstem biometry measures between patients and controls were computed (Table 3). Since cerebellar atrophy in PMM2-CDG may progress particularly in the first years of life [24], we distinguished two groups of patients based on age at the MRI: < or ≥ 36 months. On average, biometry values of cerebellar hemispheres (absolute MCD value and its ratio to MFD), cerebellar vermis (CCD and TV angle) and mid-pons were significantly decreased in PMM2-CDG group with respect to controls. Cerebellar atrophy (significantly decreased values of MCD, CCD and TV angle compared to age-matched controls) was observed in the group of patients with age < 36 months (*n*: 9; mean age 1.5 ± 1 years) as well as in the patient group with age ≥ 36 months (n: 14; mean age 11.1 ± 4.5 years). Biometry measures of mid-pons and clava were significantly decreased in the group of patients with age ≥ 36 months with respect to age-matched controls (Table 3). Functional disability (higher ADL score) was significantly associated with atrophy of cerebellar vermis (CCD) (*r*^*2*^ = 0.347; *p* = 0.004) and hemispheres (MCD) (*r*^*2*^ = 0.436; *p* = 0.005). ADL total scores were also higher in patients with a smaller brainstem owing to significantly decreased diameters of the mid-pons (*r*^*2*^ = 0.64; *p* < 0.001) the mesencephalon (*r*^*2*^ = 0.41; *p* = 0.001) and the clava (*r*^*2*^ = 0.36; *p* = 0.003) in this order.

**Table 3:** Cerebellum and brainstem MRI biometry (cm) in participants divided by age (< or ≥ 36 months)

	Patients (*n*: 23)	Controls (*n*: 19)	*p* Value	Patients < 36 mos. (*n*: 9)	Controls < 36 mos. (*n*: 8)	*p* Value	Patients ≥ 36 mos. (*n*: 14)	Controls ≥ 36 mos. (*n*: 11)	*p* Value
Age (years), mean (SD)	7.9 (6.8)	6.2 (5.4)	0.375	1.5 (1)	1.3 (1)	0.173	11.1 (4.5)	10 (3.8)	0.448
MCD	7.67 (1.0)	9.25 (1.09)	< 0.001^a^	6.92 (0.85)	8.14 (0.84)	0.026^a^	7.93 (0.94)	9.90 (0.56)	0.001^a^
CCD	2.34 (0.79)	4.18 (0.45)	< 0.001^a^	2.13 (0.25)	3.76 (0.41)	< 0.001^a^	2.42 (0.90)	4.43 (0.26)	0.001^a^
TVA	18.7 (6.61)	9.99 (3.36)	< 0.001^a^	14.8 (3.20)	10.2 (5.2)	0.076	20.1 (6.60)	9.88 (3.58)	0.001^a^
Mesencephalon	1.01 (0.23)	1.02 (0.15)	0.885	0.89 (0.10)	0.90 (0.12)	0.876	1.05 (0.25)	1.08 (0.11)	0.670
Mid-pons	1.64 (0.29)	1.92 (0.24)	0.001^a^	1.53 (0.20)	1.69 (0.16)	0.159	1.68 (0.31)	2.06 (0.17)	0.001^a^
Medulla	1.03 (0.19)	1.10 (0.18)	0.196	0.96 (0.13)	0.90 (0.10)	0.646	1.05 (0.20)	1.17 (0.18)	0.127
Clava	1.09 (0.10)	1.15 (0.15)	0.103	1.04 (0.03)	1.01 (0.09)	0.394	1.10 (0.11)	1.24 (0.11)	0.005^a^

*Age* age at MRI, *MCD* maximum transverse cerebellar diameter, *CCD* maximum craniocaudal cerebellar diameter, *TVA* tegmento-vermian angle. ^a^Statistically significant

In six subjects, MRI biometry was performed at different times during the disease course, and a total of 14 MRI were studied. Control biometry values for age at MRI examination were established using MRI’s of healthy brains from the Catania University hospital neuroimaging database. At first examination, three children aged 2–3 years showed significantly decreased MCD (7.29 ± 0.29 cm) with respect to age-matched controls (8.30 ± 0.79) (*p* = 0.037). Follow-up MRI was carried out during 1 to 6 years after the initial MRI (mean age at last MRI: 5 ± 2.6 years). On average, evolution of the measurements in time was significantly lower (7.42 ± 0.32) compared to normal controls (10.3 ± 0.96) (*p* = 0.0007). At last MRI study, total ADL scored between 18 and 29 with higher scores in those patients with more reduced MCD. One other child had a normal MCD at age 24 months (8.47; n.v. 8.30 ± 0.79). At the age 4 years, MCD was lower than the value expected for his age (8.84; n.v. 9.86 ± 0.56). This child scored 14 on ADL, and he walked independently. In two adolescent sisters aged 9 and 12 at first MRI, follow-up MRI biometry documented no significant progression of moderate cerebellar atrophy after 48 months.

**Fig. 2:**
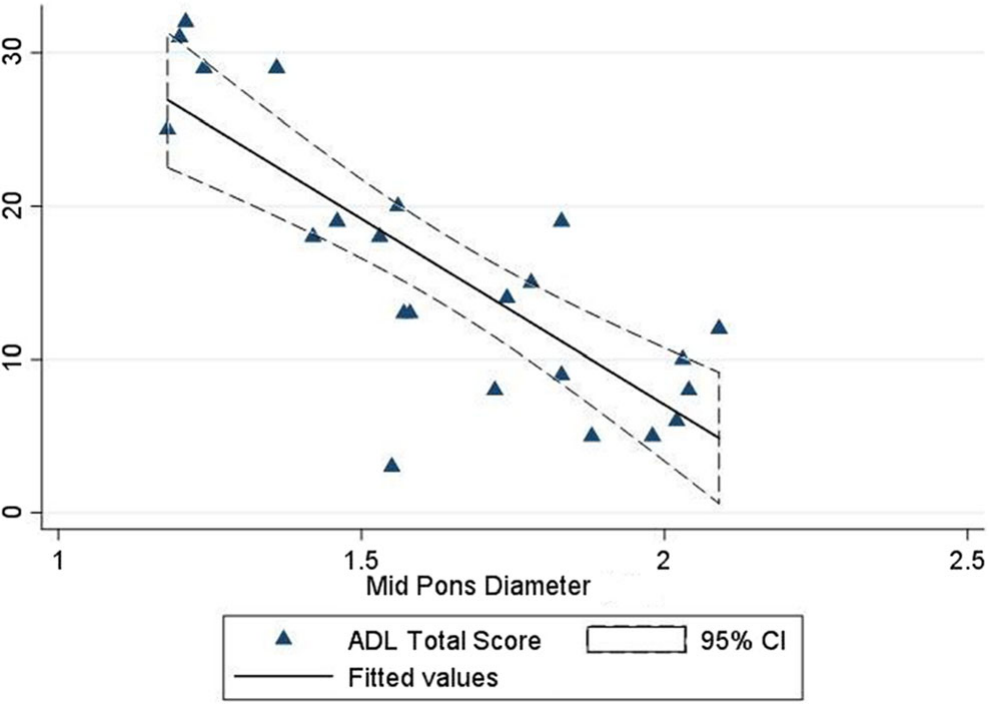
Scatter plot and regression fit line of ADL total score based on MRI measures of mid-pons diameter (cm)

MRI biometry measures which significantly correlated with total ADL score in the patients’ group (specifically: MCD, mid-pons, CCD, mesencephalon and clava) were inserted into a multiple linear regression model and selected by a stepwise backward selection, in order to obtain major predictors of the functional outcome. Diameter (cm) of the mid-pons represented alone the major predictor for the patients’ ADL total score in this model (coefficient = -25.71, S.E. coefficient = 5.12, *p* < 0.001). The model showed a good correlation between observed and predicted values (*R*^*2*^ = 0.643). Statistical variation in the dependent variables accounted for by the model was not due to chance (ANOVA *F* = 25.24; *p* = 0.0002) (Fig. 2).

## Discussion

The present study was undertaken to better understand clinical and radiological correlates of functional outcome in PMM2-CDG patients. For this purpose, we evaluated patients’ capability to fulfil daily tasks on an ADL scale, a patient/reporter outcome measure. Average ADL scores indicated moderate functional disability with a wide range demonstrating a variable functional outcome in this population. In particular, eleven patients (42%) required little or no assistance for walking (ADL walking: 1–2) and had almost preserved ADL for age-appropriated self-care domains. On the other hand, 15 patients (57%) were completely dependent on their caregivers for mobility, communicating and self-care. In front of the measured functional variability, the aim of the present work was to assess possible associations between ADL and severity of the cerebellar syndrome, genotype, concurrent neurological features and degree of cerebellar atrophy on MRI.

We found that total ADL score was significantly higher in patients with higher total BARS score indicating that patients with severe ataxia presented with more severe functional disability. In recent years, different scales have been used to assess ataxia in patients with PMM2-CDG, such as the NCRS [18], the ICARS [9], and the spinocerebellar degeneration functional score (SDFS) [11]. Interestingly, ICARS scores correlated with concurrent NCRS and were higher in patients with severe genotype and severe cerebellar atrophy [9]. We confirmed that cerebellum and mid-pons measures were significantly decreased in PMM2-CDG. In patients younger than 3 years, significant cerebellar atrophy was already measurable, whereas mid-pons and clava measures were significantly decreased in older subjects with PMM2-CDG. Noteworthy, we found an inverse correlation between functional outcome and cerebellar and brainstem diameters, particularly at the mid-pons level. In our cohort, smaller mid-pons diameter was associated with worse functional abilities (higher total ADL score) after controlling for age and studied MRI parameters inserted into a multiple linear regression model. Based on these results, we suggest that in addition to cerebellar atrophy, the occurrence of brainstem atrophy should be considered among outcome predictors in patients with PMM2-CDG.

Cerebellar atrophy in PMM2-CDG is usually detected in the neonatal period or during the first months of life. Global cerebellar hypoplasia with superimposed atrophy progresses particularly during the first decade of life [6, 8, 24] and usually stabilizes in adulthood [11]. In the current study, progressive cerebellar atrophy on repeated MRI studies paralleled functional disability in four children with PMM2-CDG aged 2–3 years at first examination. Despite the small study sample, it might be argued that decreased MCD at baseline was associated with worse functional outcome (higher ADL scores). Furthermore, MRI biometry did not detect progression in two adolescent sisters during a 2-year follow-up. This is consistent with a previous study showing that the progression rate of cerebellar atrophy measured by MCD was higher in younger children with PMM2-CDG [24].

Pontocerebellar hypoplasia (PCH) and (olivo)pontocerebellar atrophy have been described in PMM2-CDG, particularly in the more severely affected patients [8, 9, 11, 23] also at autopsy [25–27]. Depletion of Purkinje cells and granule cells [25, 28, 29] and a great loss of neurons and gliosis of dentate nuclei, olivary nuclei and pontine nuclei [25, 26, 28, 30] are hallmark pathological features of PMM2-CDG. Global PCH with superimposed atrophy and cerebellar cortex brightness on FLAIR are characteristics of PMM2-CDG among disorders with PCH-like imaging [31]. We found that additional neurological features such as severe intellectual disability and the presence of peripheral neuropathy were associated with worse functional abilities in patients with PMM2-CDG. Patients with severe ID had significantly more severe functional impairment, as expected. Developmental disability ranging from mild to severe is an almost constant feature of PMM2-CDG. However, a minority of subjects (10%) show normal cognitive development with full scale IQ scores ranging from borderline to average [2]. These patients, reported as having some degree of cerebellar atrophy, were attending regular school programs or managing to function independently in the society [32, 33]. The present study adds to this issue showing that in addition to cerebellar syndrome, cognitive function level is a major determinant of functional status in patients with PMM2-CDG.

Peripheral neuropathy occurs in almost 50 of patients with PMM2-CDG causing abolished osteotendinous reflexes, distal atrophy and pes planus [2, 7]. It contributes to muscle weakening and atrophy and impaired motor ability in PMM2-CDG [3] as observed also in the present patients. Total ADL scores were higher in participants with seizures than in those without seizures but with a non-significant difference. Generalized epilepsy, myoclonic fits and/or partial seizures have been reported from 15 to 47.8% of patients with PMM2-CDG especially in those more severely affected [7, 8, 11] supporting the association with a worst functional outcome. Functional disability measured by ADL was related to more severe cerebellar atrophy and to the presence of seizures in forty-four children with cerebellar atrophy independent of the underlying diagnosis [16]. Finally, we found a positive association between superior functional outcome and occurrence of mild *PMM2* allelic variants. In line with this observation, preserved ambulatory ability, borderline-normal intelligence [8, 32, 33] and/or isolated hand tremors and cerebellar atrophy [34] have been reported in patients with milder allelic variants such as L32R, C241S, H218L and T237M [17, 35].

Currently, there is no effective treatment for PMM2-CDG. In recent years, preclinical studies showed promising therapeutic opportunities mostly based on chaperones, liposomal mannose-1-phosphate supplementation and repurposed drugs [14]. In this context, the search for accurate outcome measures is essential. Abilities of daily living measures are patient-centred measures particularly important to assess health-related quality of life [36]. The present work highlights that studied ADL measures correlate with disease severity (NCRS), ataxia and other pertinent neurological features, thus supporting the use of ADL measures in future clinical trials.

### Limitations

Among limitations of the present study is that no specific ADL scale has been designed and validated for patients with CDG to date. The activity of daily living assessment score used in the present study was taken from the Friedreich Ataxia Rating Scale [15] and was previously validated also in children with congenital cerebellar atrophy [16]. However, this scale might not be the most appropriate for PMM2-CDG young children with a highly multisystem disorder considering that Friedreich ataxia usually presents in older children/teenagers with a different multisystem involvement. Notwithstanding, we found a good correlation between total ADL and NCRS score, a validated scale designed for patients with CDG, suggesting possible applications of ADL in PMM2-CDG. Second, most examined data were cross-sectional collected. Additional research is required to define the sensitivity and reliability of ADL in PMM2-CDG and to define clinical predictors of disease outcome prospectively.

## Conclusion

We evaluated for the first time the capability to fulfil daily tasks on an ADL scale in PMM2-CDG, a rare disease with congenital cerebellar atrophy. We found, on average, moderate functional disability with a wide range indicating a variable functional outcome. We evaluated severity of disease phenotype and cerebellar syndrome by standardized measures such as Nijmegen CDG severity scores and BARS, and we show for the first time that all these measures are associated with ADL used to quantify disease-related deficits. The occurrence of lower cognitive functioning as well as peripheral neuropathy were also significantly associated with functional disability. Finally, we show that smaller mid-pons diameter was associated with worse functional abilities after controlling for age and studied MRI parameters inserted into a multiple linear regression model. Based on these results, the present study highlights that cerebellar syndrome severity, cognitive level and peripheral neuropathy were major clinical correlates of functional disability in the studied PMM2-CDG patients. In addition, brainstem involvement should be regarded among functional outcome predictors in patients with cerebellar atrophy caused by PMM2-CDG.

## Data Availability

The anonymised data of each patient are available with unique alphanumeric code that will be shared if required by the authors.
